# Genetic associations of *Neat1* polymorphisms with clinicopathologic characteristics of tongue cancer

**DOI:** 10.7150/ijms.103842

**Published:** 2025-02-18

**Authors:** Kwei-Jing Chen, Chun-Yi Chuang, Ming-Yu Lien, Chun-Wen Su, Michael Yuan-Chien Chen, Hsiao-Chi Tsai, Shun-Fa Yang, Chih-Hsin Tang

**Affiliations:** 1 School of Dentistry, China Medical University, Taichung, Taiwan.; 2 Department of Dentistry, China Medical University Hospital, Taichung, Taiwan.; 3 School of Medicine, Chung Shan Medical University, Taichung, Taiwan.; 4 Department of Otolaryngology, Chung Shan Medical University Hospital, Taichung, Taiwan.; 5 School of Medicine, China Medical University, Taichung, Taiwan.; 6 Division of Hematology and Oncology, Department of Internal Medicine, China Medical University Hospital, Taichung, Taiwan.; 7 Institute of Medicine, Chung Shan Medical University, Taichung, Taiwan.; 8 Department of Medical Research, Chung Shan Medical University Hospital, Taichung, Taiwan.; 9 Department of Medicine Research, China Medical University Beigang Hospital, Yunlin, Taiwan.; 10 Department of Pharmacology, School of Medicine, China Medical University, Taichung, Taiwan.; 11 Department of Medical Laboratory Science and Biotechnology, Asia University, Taichung, Taiwan.; 12 Chinese Medicine Research Center, China Medical University, Taichung, Taiwan.

**Keywords:** Tongue cancer, Single nucleotide polymorphism, Neat1, lncRNAs

## Abstract

One of the most common malignant tumors of the head and neck region is tongue cancer. Long noncoding RNAs (lncRNAs) called nuclear enriched abundant transcript 1 (Neat1) are linked to tumor growth, survival, and apoptosis in a variety of cancer types; however, it is unclear how these factors relate to tongue cancer. Furthermore, it is unknown how *Neat1* polymorphisms and clinicopathological traits in individuals with tongue cancer relate to one another. We looked examined the effects of three variants in the *Neat1* gene and clinicopathological characteristics on the risk of tongue cancer in 400 male Taiwanese patients who already had the disease. Carriers of the CT+TT heterozygote of SNP rs3825071 were at a significantly lower risk to clinical stage (III+IV) and lymph node metastasis compared to those with the CC genotype. For non-smoking tongue cancer patients, but not those who smoke, the SNP rs3825071 was associated with a lower clinical stage (III+IV) and reduced lymph node metastasis. The Cancer Genome Atlas database noted that *Neat1* mRNA levels are higher in tongue cancer patients compared to normal tissues and are associated with tumor stage and metastasis. This study is the first to establish a link between the clinicopathological features of tongue cancer patients and *Neat1* polymorphisms.

## Introduction

Fifty to sixty percent of oral cancers are caused by tongue cancer, which is one of the most prevalent types of head and neck cancer 1, 2. While tongue cancer seems to be steadily rising among young adults, oral cancer is primarily found in those over the age of 55 3. Even with the advancements in recent decades in both cancer diagnosis and treatments, tongue cancer recurrence is frequent and the prognosis is still not good 4. For tongue cancer, surgery and perioperative radiation are the most often used treatments 5. Furthermore, lengthy surgery to remove the majority of the tongue at a later time harms the tongue's appearance and functionalities 5. Radiation therapy is a very successful treatment option for the majority of tongue cancer patients, regardless of the stage of the disease, and it significantly lowers the death rate of tongue cancer patients 6.

Transcripts with more than 200 nucleotides that do not have the ability to code for proteins are known as long non-coding RNAs, or lncRNAs 7-9. They have been demonstrated to be essential regulators of a number of gene expression patterns and biological processes, such as the advancement of the cell cycle and carcinogenesis 7, 8. LncRNAs affect how cells proliferate, differentiate, and die off into healthy or diseased tissues 10, 11. LncRNAs have been linked to treatment resistance and the advancement of cancer, according to a number of studies 10, 12, 13. Moreover, lncRNA may function as competitive endogenous RNAs to modify target mRNA expression by influencing miRNA expression 9. Numerous cancers have been shown to dysregulate hundreds of lncRNAs, which have thus become oncogenes or tumor-suppressors 14.

It has been observed that lncRNA nuclear enriched abundant transcript 1 (Neat1) is a crucial nuclear component that, when pulled down, causes paraspeckles to break down 15. Neat1 is linked to several human cancers, including thyroid, pancreatic, hepatic, and breast cancers 16-19. In a cell culture system, Neat1 expression was shown to be decreased in oral cancer cell lines compared to normal cells 20. In tongue cancer, researchers have investigated diagnostic biomarkers such as single nucleotide polymorphisms (SNPs) for early tongue cancer identification, precise therapy response prediction, and patient prognosis. The *Neat1* SNP has been reported to be associated with the survival of oral cancer patients 21. Our objective was to determine the associations between four *Neat1* gene SNPs and clinicopathological traits linked to Taiwanese tongue cancer risk. This work, in our opinion, shows a significant association between *Neat1* polymorphisms and tongue cancer in the Taiwanese population.

## Materials and Methods

### Participants

The Chung Shan Medical University Hospital's institutional review board in Taichung, Taiwan, gave its approval for this study (CS1-21151). This study involved 400 male patients diagnosed with tongue cancer and 1192 cancer-free controls, who did not report a history of cancer or any oral precancerous symptoms, to evaluate the influence of *Neat1* variations on the development of tongue cancer. Upon enrollment, all participants who were recruited between 2012 and 2022 provided written informed permission. The American Joint Committee on Cancer (AJCC) TNM staging approach was used to grade and stage cancer 22. Males in the control group did not disclose a history of oral precancerous diseases such as verrucous hyperplasia, erythroplakia, leukoplakia, or oral submucous fibrosis. All participants' ages and environmental risk data, such as their usage of alcohol, tobacco, and areca nuts, were collected.

### Genomic DNA extraction and PCR genotyping

Following the manufacturer's instructions, genomic DNA was extracted from peripheral blood using a QIAamp DNA Blood Kit (Qiagen, CA, USA) 23. Allelic discrimination for *Neat1* SNPs was examined in accordance with the manufacturer's instructions, as previously reported 24-26. For the examination, three *Neat1* SNPs—rs3825071, rs3741384, and rs512715—were selected. The selected criteria for three *Neat1* SNPs were all met the following two requirements: (1) according to the 1000 Genomes Project Phase 3, minor allele frequency (MAF) ≥ 0.05 in CHB population; (2) r2 for linkage disequilibrium< 0.8.

### Analysis of clinical dataset

We used an additional analysis to selected tongue cancer patients from The Cancer Genome Atlas (TCGA). Levels of *Neat1* in tongue cancer samples collected from TCGA were analyzed 27, identifying tongue cancer patients whose *Neat1* gene expression was measured in each tumor sample. The GTEx portal (gtexportal.org/home/) is a comprehensive public resource used to research tissue-specific gene expression and regulation. It offers open-access gene expression data, histology images, and quantitative trait loci (QTLs) 28.

### Statistical analysis

The statistical software package Statistical Analytic System version 9.1 (SAS Institute Inc., Cary, NC, USA) was used to analyze the data. Using the Mann-Whitney U-test, demographics and environmental exposures were compared between the patient and control groups. Genotypic ratios were compared to clinical state or tongue cancer risk using multiple logistic regression models, with potential confounders adjusted for. Between-group differences were considered significant when *p*-values were <0.05.

## Results

This study enrolled 1192 individuals who were cancer-free and 400 Taiwanese male patients who had tongue cancer in order to investigate the connection between *Neat1* polymorphisms and the emergence of tongue carcinogenesis. Both cohorts' clinical and demographic traits were evaluated (Table 1). There was no discernible age difference between the groups. But compared to the control group, a much greater percentage of tongue cancer patients smoked cigarettes, drank alcohol, and chewed betel quid (Table 1).

The genotyping data for each of the three *Neat1* SNPs for the whole research population are shown in Table 2. None of the genotypes for the three *Neat1* SNPs in the various groups shown significant relationships after controlling for alcohol consumption, cigarette smoking, and betel quid chewing (Table 2).

The adjusted odds ratio (AOR) with their 95% confidence intervals were estimated by multiple logistic regression models after controlling for betel quid chewing, cigarette smoking, and alcohol drinking.

Next, we examined the role of *Neat1* gene polymorphisms on clinicopathologic characteristics of tongue cancer patients. Compared with having the CC genotype at rs3825071, having the CT+TT heterozygote markedly lowered the risk to clinical stage III+IV (OR 0.510; 95% CI, 0.326-0.798; *p*<0.05) and lymph node metastasis (OR 0.490; 95% CI, 0.302-0.796; *p*<0.05) (Table 3). On the other hand, no significant differences were found for SNPs rs3741384 and rs512715 in relation to the clinical status of tongue cancer patients (Table 4). Furthermore, among non-smoking tongue cancer patients with the SNP rs3825071, having the CT+TT heterozygote was associated with a lower likelihood to clinical stage III+IV (OR 0.239; 95% CI, 0.097-0.591; *p*<0.05) and lymph node metastasis (OR 0.258; 95% CI, 0.100-0.666; *p*<0.05) compared to the CC wild-type (Table 5).

**Figure 1:**
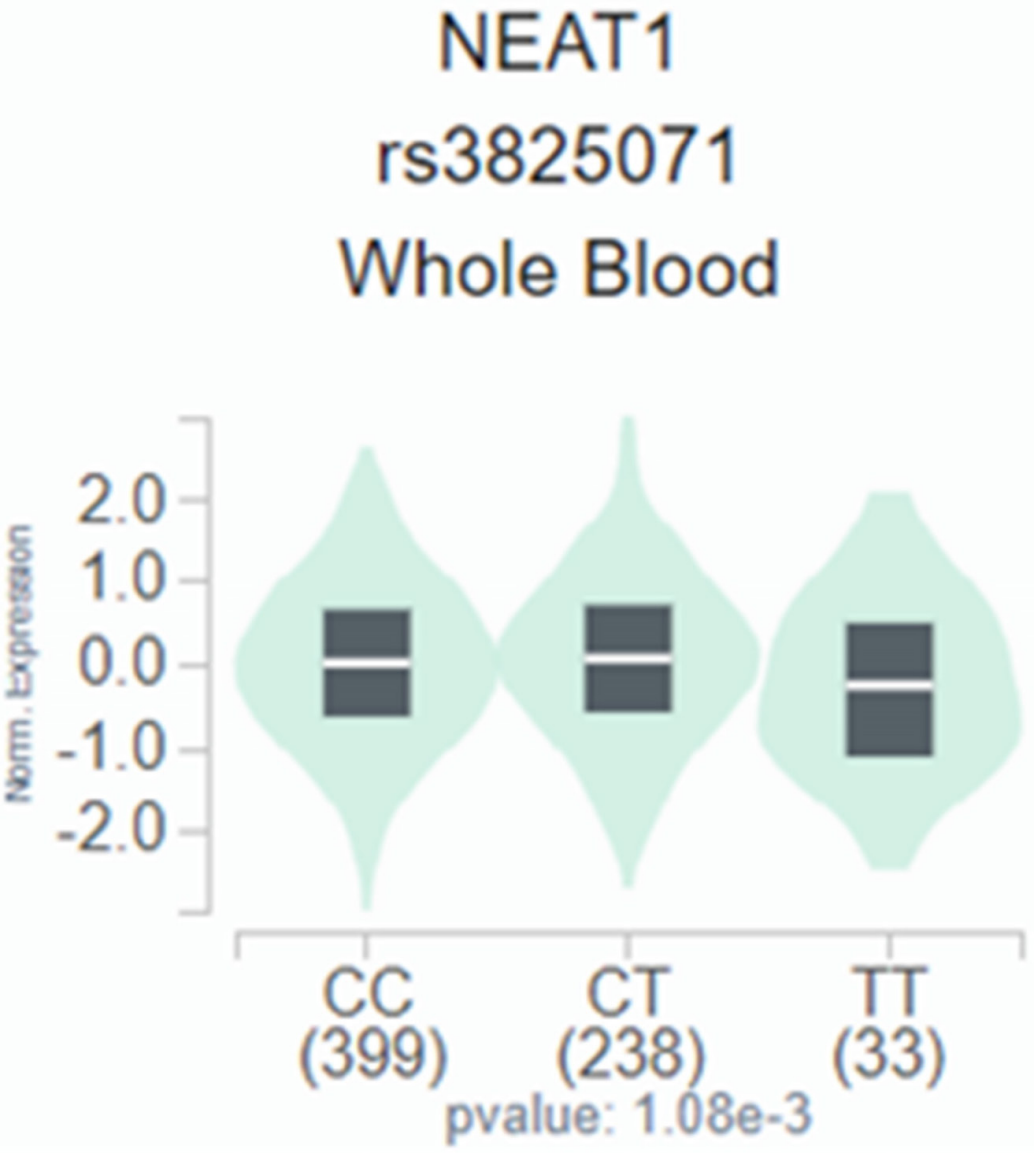
The *Neat1* presents a significant eQTL association with rs3825071 genotypes in whole blood from GTEx database.

**Figure 2:**
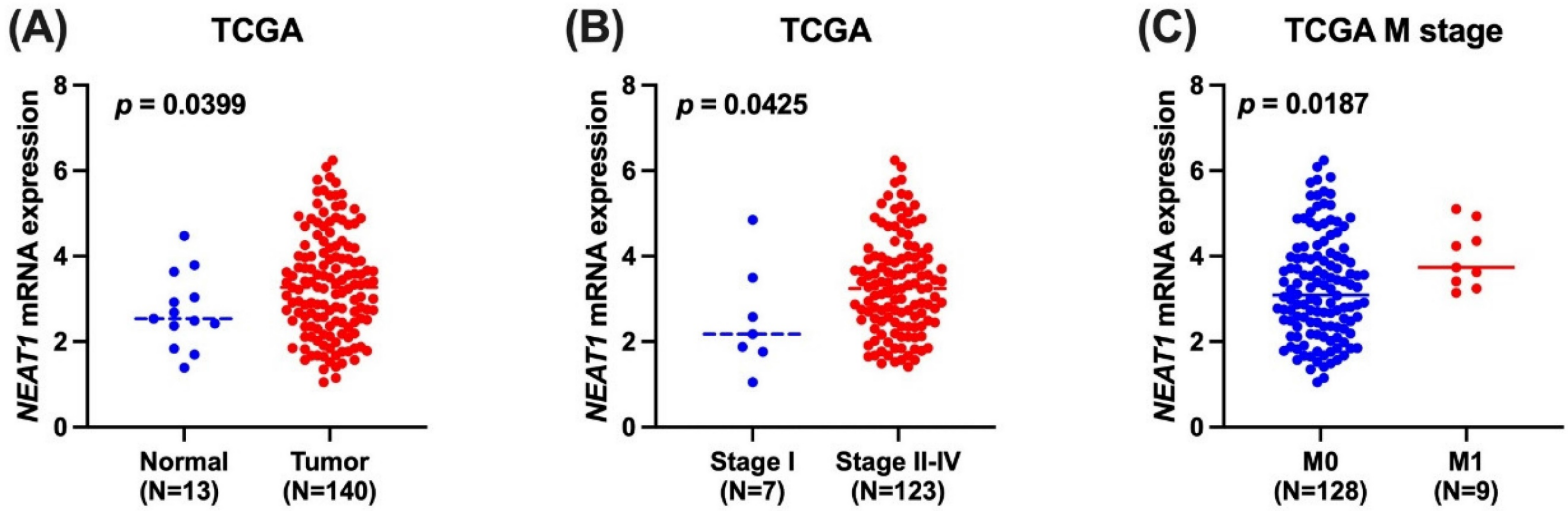
** The *Neat1* mRNA level of tongue cancer patients from TCGA database.** (A) Calculated at 95% confidence level, and (B&C) levels of *Neat1* mRNA expression retrieved from TCGA dataset records.

We employed publicly available bioinformatics datasets to confirm our findings. Neat1 expression in whole blood was shown to be lower in the rs3825071 mutation expression (CT+TT) in the GTEx database when compared to the *Neat1* allele normal type (CC) (Figure 1). We next used the TCGA database to analyze *Neat1* mRNA levels, tumor stage, and metastasis in tongue cancer patients. At a 95% confidence level, *Neat1* expression was found to be higher in tumor tissues compared to normal tissues (Figure 2A). Within tumor tissues, *Neat1* expression levels were significantly higher in patients with high-stage tumors (stages II-IV) compared to those with low-stage tumors (stage I) (Figure 2B). Additionally, *Neat1* expression was elevated in patients with metastasis (M1) (Figure 2C).

## Discussion

One of the most common malignant tumors in the head and neck region is tongue squamous cell carcinoma, which is known to behave more aggressively than traditional squamous cell carcinoma 1, 29. It is widely accepted that genetic characteristics may play a role in the tumor's heterogeneous progression. Developing a gene signature is a reliable and valuable method for patient screening, aiding in clinical decision-making 30, 31. In the current study, carriers of the CT+TT heterozygote of SNP rs3825071 were at a significantly lower risk to clinical stage III+IV and lymph node metastasis compared to those with the CC genotype. Additionally, non-smoking tongue cancer patients, unlike their smoking counterparts, exhibited similar characteristics. The *Neat1* SNP rs3825071 may represent a potential biomarker for the clinical pathogenesis of tongue cancer.

There is growing evidence that many solid tumors exhibit dysregulation of Neat1. Compared to surrounding normal tissues, kidney cell carcinoma, stomach adenocarcinoma, liver cancer, and prostate cancer all show overexpression of Neat1 32. On the other hand, pheochromocytoma, hematological malignancies, esophageal carcinomas, and breast cancer may exhibit underexpression 32. Neat1 was markedly downregulated in oral cancer patients from the Taiwanese population 20. However, Neat1 is upregulated in oral cancer tissue from patients in China, according to available literatures 33, 34. Therefore, Neat1 expression varies significantly across different cancer types. In the current human tongue cancer investigation using the TCGA database, *Neat1* expression was found to be higher in tumor tissues compared to normal tissues. Additionally, *Neat1* expression levels were upregulated in patients with high-stage tumors. Interestingly, results from the TCGA database also revealed that *Neat1* levels are associated with tumor metastasis. It has been reported that the rs3825071 variant may alter the local folding structures of *Neat1* and reduce a binding site for hsa-miR-5092 in gastric cancer patients 35. Whether the same regulatory mechanism occurs in tongue cancer requires further investigation. This lack of distinction might be attributed to the brief follow-up period and relatively small sample size. Further research is necessary, requiring an increase in both sample size and follow-up duration. Additionally, validating the current findings in an independent cohort comprising tongue cancer cases from Asian populations, as well as other cohorts available in open-access databases, is also necessary.

The process by which the initial tumor spreads to different tissues or organs through the blood or lymphatic system is referred to as "tumor metastasis" 36. Considering that inhibiting lymphangiogenesis might successfully prevent tumor growth and metastasis 37-39, it is critical to understand the complex process of metastasis. Numerous academic papers have established a connection between lymphangiogenesis and both tumor growth and metastasis 39-41. Our results indicated that the *Neat1* SNP rs3825071, with the CT+TT heterozygote, significantly lowered the risk of lymph node metastasis. Non-smoking tongue cancer patients with the SNP rs3825071 showed similar results, with an association to reduced lymph node metastasis. On the other hand, the interactions between the *Neat1* SNPs rs512715 and rs2239895 and cigarette smoking were not statistically significant in lung cancer patients 42. Cigarette smoke may influence Neat1 functions, resulting in the protective effects of *Neat1* SNPs being observed in non-smoking but not in smoking tongue cancer patients.

In conclusion, our examination demonstrates associations between *Neat1* gene variants and clinical stage as well as lymph node metastasis in tongue cancer patients, particularly among Taiwanese males carrying the *Neat1* rs3825071 polymorphism. Additionally, *Neat1* mRNA levels are higher in tongue cancer patients compared to normal tissues. This study establishes a link between the clinicopathological features of tongue cancer patients and *Neat1* polymorphisms.

## Figures and Tables

**Table 1:** The distributions of demographical characteristics in 1192 controls and 400 male patients with tongue cancer.

Variable	Controls (N=1192)	Patients (N=400)	p value
Age (yrs)			
< 60	775 (65.0%)	255 (63.8%)	p = 0.646
≥ 60	417 (35.0%)	145 (36.2%)	
Betel quid chewing			
No	995 (83.5%)	134 (33.5%)	
Yes	197 (16.5%)	266 (66.5%)	p < 0.001*
Cigarette smoking			
No	558 (46.8%)	91 (22.7%)	
Yes	634 (53.2%)	309 (77.3%)	p < 0.001*
Alcohol drinking			
No	955 (80.1%)	248 (62.0%)	
Yes	237 (19.9%)	152 (38.0%)	p < 0.001*
Stage			
I+II		181 (45.2%)	
III+IV		219 (54.8%)	
Tumor T status			
T1+T2		202 (50.5%)	
T3+T4		198 (49.5%)	
Lymph node status			
N0		246 (61.5%)	
N1+N2+N3		154 (38.5%)	
Metastasis			
M0		398 (99.5%)	
M1		2 (0.5%)	
Cell differentiation			
Well differentiated		48 (12.0%)	
Moderately or poorly differentiated		352 (88.0%)	

* p value < 0.05 as statistically significant.

**Table 2:** Odds ratio (OR) and 95% confidence interval (CI) of tongue cancer associated with *Neat1* genotypic frequencies.

Variable	Controls (N=1192) (%)	Patients (N=400) (%)	AOR (95% CI)	p value
**rs3825071**				
CC	830 (69.6%)	292 (73.0%)	1.000 (reference)	
CT	324 (27.2%)	95 (23.8%)	0.898 (0.666-1.213)	p=0.485
TT	38 (3.2%)	13 (3.2%)	0.940 (0.451-1.957)	p=0.869
CT+TT	362 (30.4%)	108 (27.0%)	0.903 (0.678-1.203)	p=0.487
**rs3741384**				
GG	848 (71.1%)	290 (72.5%)	1.000 (reference)	
GA	312 (26.2%)	101 (25.3%)	0.999 (0.742-1.344)	p=0.993
AA	32 (2.7%)	9 (2.2%)	0.861 (0.369-2.009)	p=0.729
GA+AA	344 (28.9%)	110 (27.5%)	0.986 (0.740-1.314)	p=0.922
**rs512715**				
GG	652 (54.7%)	215 (53.8%)	1.000 (reference)	
GC	468 (39.3%)	167 (41.8%)	1.122 (0.858-1.466)	p=0.401
CC	72 (6.0%)	18 (4.4%)	0.743 (0.406-1.358)	p=0.334
GC+CC	540 (45.3%)	185 (46.2%)	1.069 (0.825-1.385)	p=0.615

**Table 3:** Odds ratio (OR) and 95% confidence intervals (CI) of clinical statuses associated with genotypic frequencies of *Neat1* rs3825071 in 400 male tongue cancer patients.

Variable	CC (N=292)	CT+TT (N=108)	OR (95% CI)	p value
**Clinical Stage**				
Stage I+II	119 (40.8%)	62 (57.4%)	1.000 (reference)	p=0.003*
Stage III+IV	173 (59.2%)	46 (42.6%)	**0.510 (0.326-0.798)**	
**Tumor size**				
≦ T2	144 (49.3%)	58 (53.7%)	1.000 (reference)	p=0.436
> T2	148 (50.7%)	50 (46.3%)	0.839 (0.539-1.305)	
**Lymph node metastasis**				
No	167 (57.2%)	79 (73.1%)	1.000 (reference)	p=0.004*
Yes	125 (42.8%)	29 (26.9%)	**0.490 (0.302-0.796)**	
**Metastasis**				
M0	290 (99.3%)	108 (100.0%)	1.000 (reference)	---
M1	2 (0.7%)	0 (0.0%)	---	
**Cell differentiated grade**				
Well	33 (11.3%)	15 (13.9%)	1.000 (reference)	p=0.480
Moderate or poor	259 (88.7%)	93 (86.1%)	0.790 (0.410-1.520)	

The odds ratio (OR) with their 95% confidence intervals were estimated by logistic regression models. * p value < 0.05 as statistically significant.

**Table 4:** Clinical statuses and genotypic frequencies of *Neat1* rs3741384 and rs512715 in 400 male tongue cancer patients.

	rs3741384				rs512715			
Variable	GG (N=290)	GA+AA (N=110)	OR (95% CI)	p value	GG (N=215)	GC+CC (N=185)	OR (95% CI)	p value
**Clinical Stage**								
Stage I+II	133 (45.9%)	48 (43.6%)	1.000 (reference)	0.690	101 (47.0%)	80 (43.2%)	1.000 (reference)	0.454
Stage III+IV	157 (54.1%)	62 (56.4%)	1.094 (0.703-1.702)		114 (53.0%)	105 (56.8%)	1.163 (0.783-1.727)	
**Tumor size**								
≦ T2	151 (52.1%)	51 (46.4%)	1.000 (reference)	0.308	118 (54.9%)	84 (45.4%)	1.000 (reference)	0.059
> T2	139 (47.9%)	59 (53.6%)	1.257 (0.809-1.951)		97 (45.1%)	101 (54.6%)	1.463 (0.986-2.171)	
**Lymph node metastasis**								
No	186 (64.1%)	60 (54.5%)	1.000 (reference)	0.078	139 (64.7%)	107 (57.8%)	1.000 (reference)	0.163
Yes	104 (35.9%)	50 (45.5%)	1.490 (0.955-2.327)		76 (35.3%)	78 (42.2%)	1.333 (0.890-1.997)	
**Metastasis**								
M0	288 (99.3%)	110 (100%)	1.000 (reference)	---	213 (99.1%)	185 (100%)	1.000 (reference)	---
M1	2 (0.7%)	0 (0.0%)	---		2 (0.9%)	0 (0.0%)	---	
**Cell differentiation**								
Well	31 (10.7%)	17 (15.5%)	1.000 (reference)	0.190	24 (11.2%)	24 (13.0%)	1.000 (reference)	0.579
Moderate or poor	259 (89.3%)	93 (84.5%)	0.655 (0.346-1.238)		191 (88.8%)	161 (87.0%)	0.843 (0.461-1.541)	

The odds ratio (OR) with their 95% confidence intervals were estimated by logistic regression models.

**Table 5:** Clinical statuses and genotypic frequencies of *Neat1* rs3825071 in 400 male tongue cancer patients who are smoker and non-smokers.

	Non-smoker (N=91)				smoker (N=309)			
Variable	CC (N=57)	CT+TT (N=34)	OR (95% CI)	p value	CC (N=235)	CT+TT (N=74)	OR (95% CI)	p value
**Clinical Stage**								
Stage I+II	19 (33.3%)	23 (67.6%)	1.000 (reference)	0.001*	100 (42.6%)	39 (52.7%)	1.000 (reference)	0.126
Stage III+IV	38 (66.7%)	11 (32.4%)	**0.239 (0.097-0.591)**		135 (57.4%)	35 (47.3%)	0.665 (0.393-1.123)	
**Tumor size**								
≦ T2	26 (45.6%)	19 (55.9%)	1.000 (reference)	0.343	118 (50.2%)	39 (52.7%)	1.000 (reference)	0.709
> T2	31 (54.4%)	15 (44.1%)	0.662 (0.282-1.556)		117 (49.8%)	35 (47.3%)	0.905 (0.536-1.527)	
**Lymph node metastasis**								
No	26 (45.6%)	26 (76.5%)	1.000 (reference)	0.004*	141 (60.0%)	53 (71.6%)	1.000 (reference)	0.071
Yes	31 (54.4%)	8 (23.5%)	**0.258 (0.100-0.666)**		94 (40.0%)	21 (28.4%)	0.594 (0.337-1.050)	
**Metastasis**								
M0	56 (98.2%)	34 (100.0%)	1.000 (reference)	---	234 (99.6%)	74 (100.0%)	1.000 (reference)	---
M1	1 (1.8%)	0 (0.0%)	---		1 (0.4%)	0 (0.0%)	---	
**Cell differentiation**								
Well	4 (7.0%)	6 (17.6%)	1.000 (reference)	0.117	29 (12.3%)	9 (12.2%)	1.000 (reference)	0.968
Moderate or poor	53 (93.0%)	28 (82.4%)	0.352 (0.092-1.352)		206 (87.7%)	65 (87.8%)	1.017 (0.458-2.259)	

The odds ratio (OR) with their 95% confidence intervals were estimated by logistic regression models. * p value < 0.05 as statistically significant.

## References

[1] Matsuo K, Akiba J, Kusukawa J, Yano H (2022). Squamous cell carcinoma of the tongue: subtypes and morphological features affecting prognosis. Am J Physiol Cell Physiol.

[2] Shahhosseini A, Bourova-Flin E, Derakhshan S, Aminishakib P, Goudarzi A (2023). High levels of histone H3 K27 acetylation and tri-methylation are associated with shorter survival in oral squamous cell carcinoma patients. BioMedicine.

[3] Paderno A, Morello R, Piazza C (2018). Tongue carcinoma in young adults: a review of the literature. Acta Otorhinolaryngol Ital.

[4] da Silva Souto AC, Vieira Heimlich F, Lima de Oliveira L, Bergmann A, Dias FL, Spindola Antunes H (2023). Epidemiology of tongue squamous cell carcinoma: A retrospective cohort study. Oral Dis.

[5] Lim YJ, Kong M (2021). Population-based comparative survival analysis of surgery with or without adjuvant radiotherapy and non-operative primary radiotherapy in patients with early-stage oral tongue squamous cell carcinoma. PloS one.

[6] Hyytiäinen A, Mroueh R, Peltonen J, Wennerstrand P, Mäkitie A, Al-Samadi A (2023). Prognostic histological markers in oral tongue squamous cell carcinoma patients treated with (chemo)radiotherapy. APMIS: acta pathologica, microbiologica, et immunologica Scandinavica.

[7] Forrest ME, Khalil AM (2017). Review: Regulation of the cancer epigenome by long non-coding RNAs. Cancer Lett.

[8] Paralkar VR, Weiss MJ (2013). Long noncoding RNAs in biology and hematopoiesis. Blood.

[9] Wang YH, Tsai CH, Liu SC, Chen HT, Chang JW, Ko CY (2022). miR-150-5p and XIST interaction controls monocyte adherence: Implications for osteoarthritis therapy. Front Immunol.

[10] Ulitsky I, Bartel DP (2013). lincRNAs: genomics, evolution, and mechanisms. Cell.

[11] Su SC, Yeh CM, Lin CW, Hsieh YH, Chuang CY, Tang CH (2021). A novel melatonin-regulated lncRNA suppresses TPA-induced oral cancer cell motility through replenishing PRUNE2 expression. J Pineal Res.

[12] Sun S, Del Rosario BC, Szanto A, Ogawa Y, Jeon Y, Lee JT (2013). Jpx RNA activates Xist by evicting CTCF. Cell.

[13] Yuan LT, Yang YC, Lee HL, Shih PC, Chen LH, Tang CH (2022). Genetic Polymorphisms of lncRNA LINC00673 as Predictors of Hepatocellular Carcinoma Progression in an Elderly Population. International journal of molecular sciences.

[14] He J, Huang B, Zhang K, Liu M, Xu T (2020). Long non-coding RNA in cervical cancer: From biology to therapeutic opportunity. Biomed Pharmacother.

[15] Yu X, Li Z, Zheng H, Chan MT, Wu WK (2017). NEAT1: A novel cancer-related long non-coding RNA. Cell Prolif.

[16] Zhang M, Wu WB, Wang ZW, Wang XH (2017). lncRNA NEAT1 is closely related with progression of breast cancer via promoting proliferation and EMT. Eur Rev Med Pharmacol Sci.

[17] Mang Y, Li L, Ran J, Zhang S, Liu J, Li L (2017). Long noncoding RNA NEAT1 promotes cell proliferation and invasion by regulating hnRNP A2 expression in hepatocellular carcinoma cells. OncoTargets and therapy.

[18] Li JH, Zhang SQ, Qiu XG, Zhang SJ, Zheng SH, Zhang DH (2017). Long non-coding RNA NEAT1 promotes malignant progression of thyroid carcinoma by regulating miRNA-214. Int J Oncol.

[19] Chakravarty D, Sboner A, Nair SS, Giannopoulou E, Li R, Hennig S (2014). The oestrogen receptor alpha-regulated lncRNA NEAT1 is a critical modulator of prostate cancer. Nature communications.

[20] Lin NC, Hsia SM, Wang TH, Li PJ, Tseng YH, Chiu KC (2022). The relation between NEAT1 expression level and survival rate in patients with oral squamous cell carcinoma. Journal of dental sciences.

[21] Zhu L, He Y, Feng G, Yu Y, Wang R, Chen N (2021). Genetic variants in long non-coding RNAs UCA1 and NEAT1 were associated with the prognosis of oral squamous cell carcinoma. International journal of oral and maxillofacial surgery.

[22] Edge SB, Compton CC (2010). The American Joint Committee on Cancer: the 7th edition of the AJCC cancer staging manual and the future of TNM. Ann Surg Oncol.

[23] Yang MD, Lin KC, Lu MC, Jeng LB, Hsiao CL, Yueh TC (2017). Contribution of matrix metalloproteinases-1 genotypes to gastric cancer susceptibility in Taiwan. Biomedicine (Taipei).

[24] Chang SL, Yang PJ, Lin YY, Jiang YJ, Liu PI, Huang CL (2022). Genetic Associations of Visfatin Polymorphisms with EGFR Status and Clinicopathologic Characteristics in Lung Adenocarcinoma. International journal of environmental research and public health.

[25] Chen KJ, Hsieh MH, Lin YY, Chen MY, Lien MY, Yang SF (2022). Visfatin Polymorphisms, Lifestyle Risk Factors and Risk of Oral Squamous Cell Carcinoma in a Cohort of Taiwanese Males. International journal of medical sciences.

[26] Li HM, Wang LJ, Tang F, Pan HF, Zhang TP (2022). Association Between Genetic Polymorphisms of lncRNA NEAT1 and Pulmonary Tuberculosis Risk, Clinical Manifestations in a Chinese Population. Infection and drug resistance.

[27] Lin TH, Chang SL, Khanh PM, Trang NTN, Liu SC, Tsai HC (2022). Apelin Promotes Prostate Cancer Metastasis by Downregulating TIMP2 via Increases in miR-106a-5p Expression. Cells.

[28] Carithers LJ, Moore HM (2015). The Genotype-Tissue Expression (GTEx) Project. Biopreserv Biobank.

[29] de Boer J, Barnett R, Cardin A, Cimoli M, Davies L, Delany C (2024). Optimising Patient Outcomes in Tongue Cancer: A Multidisciplinary Approach. Cancers.

[30] Scully C, Field JK, Tanzawa H (2000). Genetic aberrations in oral or head and neck squamous cell carcinoma (SCCHN): 1. Carcinogen metabolism, DNA repair and cell cycle control. Oral Oncol.

[31] Scully C, Field J (1997). Genetic aberrations in squamous cell carcinoma of the head and neck (SCCHN), with reference to oral carcinoma (review). Int J Oncol.

[32] Li S, Li J, Chen C, Zhang R, Wang K (2018). Pan-cancer analysis of long non-coding RNA NEAT1 in various cancers. Genes & diseases.

[33] Liu X, Shang W, Zheng F (2018). Long non-coding RNA NEAT1 promotes migration and invasion of oral squamous cell carcinoma cells by sponging microRNA-365. Exp Ther Med.

[34] Huang G, He X, Wei XL (2018). lncRNA NEAT1 promotes cell proliferation and invasion by regulating miR-365/RGS20 in oral squamous cell carcinoma. Oncol Rep.

[35] Ji X, Yan Y, Ma N, He G, Wang K, Zhang Y (2021). Variant of SNPs at lncRNA NEAT1 contributes to gastric cancer susceptibility in Chinese Han population. International journal of clinical oncology.

[36] Lei PJ, Fraser C, Jones D, Ubellacker JM, Padera TP (2024). Lymphatic system regulation of anti-cancer immunity and metastasis. Front Immunol.

[37] Dieterich LC, Detmar M (2016). Tumor lymphangiogenesis and new drug development. Adv Drug Deliv Rev.

[38] Lin CY, Wang SW, Chen YL, Chou WY, Lin TY, Chen WC (2017). Brain-derived neurotrophic factor promotes VEGF-C-dependent lymphangiogenesis by suppressing miR-624-3p in human chondrosarcoma cells. Cell Death Dis.

[39] Lee HP, Wang SW, Wu YC, Lin LW, Tsai FJ, Yang JS (2020). Soya-cerebroside inhibits VEGF-facilitated angiogenesis in endothelial progenitor cells. Food Agr Immunol.

[40] Paduch R (2016). The role of lymphangiogenesis and angiogenesis in tumor metastasis. Cell Oncol (Dordr).

[41] Su CM, Tang CH, Chi MJ, Lin CY, Fong YC, Liu YC (2018). Resistin facilitates VEGF-C-associated lymphangiogenesis by inhibiting miR-186 in human chondrosarcoma cells. Biochem Pharmacol.

[42] Wang S, Cui Z, Li H, Li J, Lv X, Yang Z (2019). LncRNA NEAT1 polymorphisms and lung cancer susceptibility in a Chinese Northeast Han Population: A case-control study. Pathology, research and practice.

